# Psychological stress of general practitioners in the care of patients with palliative care needs: an exploratory study

**DOI:** 10.1186/s12904-024-01529-w

**Published:** 2024-08-03

**Authors:** Verena Lopez, Piet van der Keylen, Thomas Kühlein, Maria Sebastião

**Affiliations:** 1 Institute of General Practice, Friedrich-Alexander-University Erlangen-Nürnberg, University Hospital Erlangen, Universitätsstr. 29, 91054 Erlangen, Germany; 2 Lutheran University of Applied Sciences, Nürnberg, Germany

**Keywords:** Palliative care, General practitioner, Stress, Stressor, Burden, Emotional, Coping mechanism, Advance care planning

## Abstract

**Background:**

In Germany, general practitioners play a pivotal role in palliative care provision. Caring for patients with palliative care needs can be a burden for general practitioners, highlighting the importance of self-care and mental health support. This study aimed to explore the role of palliative care in general practitioners’ daily work, the stressors they experience, their coping mechanisms, and the potential benefits of Advance Care Planning in this context.

**Methods:**

An exploratory approach was employed, combining a short quantitative survey with qualitative interviews. The analysis was based on a structuring qualitative content analysis, following a deductive-inductive procedure and integrating the Stress-Strain Model and Lazarus’ Transactional Model of Stress and Coping. We recruited eleven general practitioners to take part in the study.

**Results:**

General practitioners viewed palliative care as integral to their practice but faced challenges such as time constraints and perceived expertise gaps. Societal taboos often hindered conversations on the topic of death. Most general practitioners waited for their patients to initiate the topic. Some general practitioners viewed aspects of palliative care as potentially distressing. They used problem-focused (avoiding negative stressors, structuring their daily schedules) and emotion-focused (discussions with colleagues) coping strategies. Still, general practitioners indicated a desire for specific psychological support options. Advance Care Planning, though relatively unfamiliar, was acknowledged as valuable for end-of-life conversations.

**Conclusions:**

Palliative care can be associated with negative psychological stress for general practitioners, often coming from external factors. Despite individual coping strategies in place, it is advisable to explore concepts for professional psychological relief.

**Trial registration:**

Not registered.

**Supplementary Information:**

The online version contains supplementary material available at 10.1186/s12904-024-01529-w.

## Background

Palliative care is defined as “*the active holistic care of individuals across all ages with serious health-related suffering due to severe illness and especially of those near the end of life. It aims to improve the quality of life of patients*,* their families and their caregivers*” [1]. Palliative care has a positive effect on end-of-life care - for example, in reducing potentially aggressive medical interventions such as intensive medical treatment [2]. Between 69 and 82% of terminally ill patients need palliative care [3]. According to the World Health Organization (WHO) in 2020, an estimated 56.8 million people required palliative care globally. However, only about 14% of those in need received it. In the coming years, more patients will need palliative care services, primarily driven by the ageing of the population and the growing burden of diseases [4].

Palliative care can be provided in an ambulatory or an inpatient setting. In Germany, 76% of patients expressed a preference for dying at home [5] which means that general practitioners (GPs) play a central role in providing palliative care [6]. In Germany, basic knowledge of palliative medicine is part of the training requirements for becoming a GP [7]. GPs who wish to expand their expertise can participate in further training programs. Specialized Ambulatory Palliative Care Teams (SAPCT) step in when patients’ symptoms and needs exceed the capabilities of GPs.

Caring for patients with palliative care needs can become burdensome for GPs, e.g. due to time constraints [8, 9]. This lack of time is intensified by a high caseload and the fact that patients with palliative care needs often require more time and attention than other patient groups [8]. Parallels, such as similar age or character traits, may lead to reflections on their own mortality and death, presenting a negative psychological burden for GPs [10]. This burden may intensify when patients express their wish for hastening death [11, 12]. Currently, there is little information on how GPs in Germany conduct conversations about end-of-life care [13]. Advance Care Planning (ACP) as a proactive process in which patients discuss and document their preferences regarding future medical decisions could be helpful [14]. Although the financing of ACP was regulated by the Hospice and Palliative Care Act passed in 2015, the concept is not yet widely established in Germany [15]. It has been shown that ACP has positive effects on care for patients [16]. It remains to be seen whether ACP also has a positive influence on the mental health of GPs.

In Germany, legal frameworks allow treatment limitations (e.g. no artificial nutrition) and palliative care measures (e.g. sufficient pain relief despite potential shortening of life or palliative sedation) but explicitly prohibit active euthanasia [17]. Assisted suicide, involving the provision of lethal substances, exists within a legal grey area [17]. Although a 2020 ruling by the Federal Constitutional Court emphasized the right to self-determined dying, legal uncertainties remain (BVerfG, Guidelines to the Judgment of the Second Senate, February 26, 2020, Art. 2 para. 1). This uncertainty may lead to an additional burden for GPs when caring for patients with palliative care needs.

The high rates of burnout among GPs (32–43%) [18] and the high suicide rate among physicians in general [19] highlight the importance of mental health and self-care. As dealing with death can be a potential psychological stressor for GPs, attention should be directed towards this topic. Stress reactions do not inherently result in health damage [20]. The decisive factor is the interplay between the stressors, their intensity, timing, and individual resources. Therefore, this study aimed to explore (1) the role of palliative care in the daily work of GPs, (2) the psychological and emotional stress experienced by GPs in this context, (3) their possible coping mechanisms, and (4) if ACP can be supportive for the GPs’ mental health in the palliative care setting.

## Methods

### Study design

An exploratory approach with a short quantitative survey followed by a qualitative interview was chosen to understand the GPs’ perspective. The ethics committee of the Friedrich-Alexander University Erlangen-Nürnberg approved the study (file number 21-377-B).

### Recruitment and setting

The recruitment was conducted through contacts of the Institute of General Practice. We used different recruitment channels. GPs were invited to participate in the study via the Institute’s newsletter (approximately 250 recipients). We also distributed information at various events (e.g. research symposium for GPs). We asked GPs to further spread the study (snowball system). In addition, 30 randomly selected practices in Bavaria were made aware of the study by e-mail and/or telephone. Inclusion criteria were: GPs practicing in Bavaria, Germany, including doctors-in-training and GPs who retired within the last year. We planned for 15–20 interviews to be conducted. Prospective participants received an invitation letter and study information (*n* = 33). GPs expressing interest subsequently received the data protection declaration, consent form for signature, and a brief survey for completion (*n* = 11). The most frequently cited reason for non-participation was lack of time. The interviewer (VL, female, student of human medicine) and the GPs did not know each other beforehand.

### Data collection

The project team developed the interview guide. Based on a literature review, we created mind maps for each research question which depicted various influencing factors and elements. After structuring and summarizing, four thematic blocks emerged for the interview guide: (1) Experience with palliative care as a GP, (2) Patients’ death wishes and the resulting emotions on the side of the GPs, (3) Dealing with any psychological stressors, and (4) Suggestions for coping with psychological stressors in palliative care (including questions about ACP) (Supplemental file 1).

The interview guide was pre-tested with two GPs. After the pre-test, a written definition of ACP was incorporated into the interview guide, and questions related to ACP were modified. The quantitative survey comprised questions concerning socio-demographic status and a subset of 15 questions (drawn from a total of 144) sourced from the “*Questionnaire on attitudes towards dying*,* death*,* and the afterlife*” (FESTD) [21] (Supplemental file 2). Questions were chosen based on the relevance to the care of patients with palliative care needs and to gain an impression of the GPs’ attitude to dying and death. The participants received the paper questionnaire by post or email in advance and were asked to complete and return it before the interview.

From 12/2021 to 11/2022 data collection took place. VL conducted the interviews (*n* = 11) via Zoom (zoom.us, San José, USA). The interviews (35–80 min, on average 60 min) were digitally audio-recorded and later transcribed verbatim using the software f4 (Marburg, Germany). Transcripts were not provided to participants. In addition, protocols of the interviews were written to include e.g. first memos. The memos contained first ideas for the data analysis. These were later used to familiarize ourselves with the material as a first step of data analysis.

### Data analysis

**Fig. 1:**
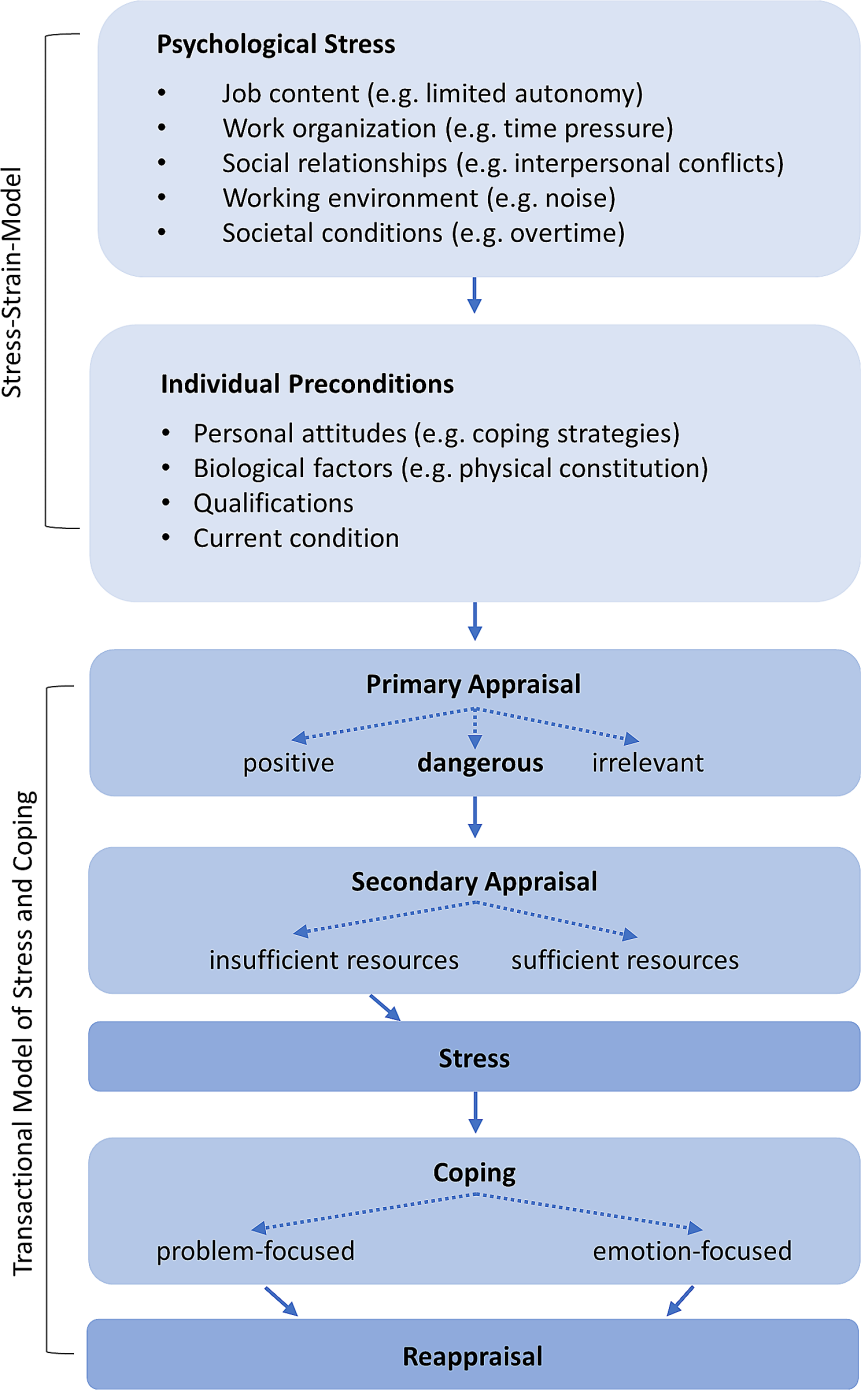
Combination of the two stress models used for analysis

As a basis for our analysis, we combined two models: 1) The *Stress-Strain-Model*, developed by Rohmert and Rutenfranz [22], and the *Transactional Model of Stress and Coping* developed by Lazarus [20]. We used these two models to form our deductive categories. The *Stress-Strain-Modell* delineates the causal relationship between mental stress and strain. The various workload influences affect the individual with different preconditions (Fig. 1).

The *Transactional Model of Stress and Coping* describes the interaction between individuals and their environment as a dynamic and reciprocally influential process [20] (Fig. 1). When an individual is confronted with a stressor, a primary appraisal takes place in which the stressor is classified as positive, irrelevant, or threatening. This means that a stressor is not necessarily associated with a threat and can also be evaluated positively. Only if a threat is perceived, a secondary appraisal follows in which the available resources are assessed. Insufficient resources lead to stress reactions.

Negative consequences arise from the mismatch between work-related demands (such as job contenct or work organization) and individual preconditions (such as personal attitude or qualification). Consequently, identical work-related stressors can lead to varying stress levels and outcomes in the short and long term for diverse individuals or on different occasions. Coping strategies become necessary to restore a balance between demands and resources. Those coping strategies can be problem-oriented (modifying the situation) or emotion-oriented (changing the attitude). Ultimately, the situation is re-evaluated.

The analysis was based on the structuring qualitative content analysis by Kuckartz and Rädiker [23], following a deductive-inductive procedure and using the software program MAXQDA Plus 2020. Initially, VL wrote a case summary for each participant. VL and MS deductively formed a system of categories based on the two stress models. The categories were provided with a short description. During analysis, VL added an example for each category. VL applied the categories to the material. The category system was inductively expanded by new categories and re-organized. After coding eight of the interviews, no new categories were added, indicating data saturation. All data material was coded using the final category system (Supplemental file 3). On an individual level, we compared answers from the questionnaire and interviews to identify discrepancies. We then checked whether these discrepancies were also found in other interviews.

### Quality assurance

TK (male, Chair of Institute, GP) and MS (female, senior researcher, Public Health) have extensive knowledge in qualitative research. Before data collection, VL was trained in qualitative data collection and analysis. All categories were continuously discussed by the project team (VL, PvK, TK, MS) meeting the standard of consensual validation. VL kept a research diary to reflect on her role and possible influence during the research process. The reporting of the study follows the “*Consolidated criteria for reporting qualitative research*” (COREQ) [24].

## Results

In our study, 11 GPs (6 men, 5 women) of various age groups participated (Table 1).

**Table 1:** Socio-demographic data and information on residency program and employment status

Characteristics	Specification	*n*
Age	30–40 Years	2
	41–50 Years	4
	51–60 Years	2
	61–70 Years	3
Gender	Female	6
	Male	5
Specialization (multiple answers possible)	General medicine	8
	General internal medicine	3
	Other	3
Employment (duration)	0–3 Years	2
	4–10 Years	2
	11–20 Years	4
	21–30 Years	1
	> 31 Years	2
Employment status	Employed	4
	Self-employed	7
Weekly work time	< 31 h	4
	31–40 h	2
	41–50 h	2
	51–60 h	2
	> 60 h	1
Type of practice	Single practice	1
	Group practice	9
	Medical care center	1
Location of practice	Rural (< 5 000 residents)	4
	Small-sized town (5 000–20 000 residents)	4
	Medium-sized town (20 000–100 000 residents)	1
	City (> 100 000 residents)	2

The presentation of the results follows the research questions, commencing with the role of palliative care (Q1), followed by an exploration of potential stressors (Q2), and coping mechanisms (Q3) and concluding with ACP (Q4).

### Role of palliative care

The central objective of GPs in palliative care is to alleviate suffering and thus maintain quality of life (GP1; GP2; GP3; GP9). Palliative care holds a high to very high significance in the daily practice of GPs. It is perceived as an “*inherent responsibility of general practice*” (GP7) to accompany patients until the end of their lives. This perception was also reflected in their professional identity: GPs saw themselves as medical companions from childhood to death. The importance was not necessarily directly proportional to the actual level of palliative care provided: “*For me*,* it is very important […]. But fortunately*,* it is a small percentage of patients*” (GP10). The average number of patients with palliative care needs cared for per year showed a wide range. Most GPs (*n* = 8) stated that they treat up to 20 patients yearly, the maximum was over 60 patients (*n* = 1). The majority (10 out of 11) stated that between 0 and 20 patients with palliative care needs die in their care over the span of one year (Supplemental information 2).

### Psychological and emotional stress experienced by GPs

#### External stressors

##### Job content

For GPs, palliative care was considered a “*routine aspect*” (GP9), however, they reported a significant imbalance between effort and reimbursement (GP4; GP7; GP9). In general, GPs expressed openness to discussing death and related wishes with their patients. Some emphasized a moral obligation to address these topics (GP5), highlighting the ethical dimension involved. However, there were clear differences in how these discussions were initiated. While some GPs actively approached patients to discuss end-of-life care (GP3; GP4; GP7; GP9), the majority of them waited for patients to raise the topic. Many GPs appreciate when patients initiate these conversations, as it signals openness to the topic and provides a starting point. The most common triggers were critical health conditions, acute deteriorations, an unfavorable prognosis (GP2; GP4; GP7; GP9) or death in the patient’s social environment (GP9; GP10). The identified triggers also determine the primary target groups for these discussions: patients with palliative care needs, chronically ill individuals, older patients, and their respective relatives, along with those who actively approach GPs regardless of age or health status (GP2; GP3; GP7; GP8; GP9; GP10; GP11). Even with a specific trigger, GPs described that they sometimes hesitate to initiate discussions to avoid overwhelming patients. GPs also expressed concerns about potentially diminishing patients’ hope and will to live through discussions about death (GP4; GP6; GP8).

Central to these conversations is the understanding of patients’ preferences and wishes (GP1; GP3; GP5; GP7; GP8). This includes evaluating whether home care is feasible and if a move to a hospice is necessary and desired. The involvement of family members in end-of-life care and their alignment with the patients’ wishes is crucial (GP9). Requests for physician-assisted suicide were mentioned less frequently, and some GPs expressed relief about that: “*many patients were so hopeless and deeply sad at the end*,* […] that they said ‘I would prefer to be dead right now.’ But I’m so glad that not a single person asked me if I could help them.”* (GP10). When no specific wishes were documented, the presumed patient will has to be determined in acute situations. This can lead to challenging situations for all parties involved (GP7; GP8).

##### Work organization

GPs expressed a fundamental time constraint, noting that “*the main part of (…) caring for dying patients is certainly a verbal one and that takes time. And the second point of course*,* is that these patients are often so ill that they need home visits*” (GP1). A certain level of flexibility is often necessary because “*palliative care is always something spontaneous and difficult to plan. I can’t really say I’ll keep ten to twelve free in my diary on Friday’ or something; that’s not really possible*” (GP6). This time challenge becomes more pronounced when patients have not made any or only insufficient decisions regarding palliative care. GPs described advance care planning as time-consuming consultations to explain medical terms and treatment options (GP5; GP10). While justified given the gravity of the decisions, these lengthy discussions pose organizational challenges for GPs (GP1; GP2).

##### Work environment

Caring at home for patients with palliative care needs is commonly favored and often aligns with the patients’ wishes. The GPs named positive impacts of the familiar environment on the psychological well-being of patients (GP1). In certain situations, such as when the nursing care needs cannot be adequately met, GPs preferred hospice care. Some interviewees explicitly assessed any demand for round-the-clock availability as a burden (GP7; GP8).

The GPs mentioned the time constraints as the most prominent obstacle in the ambulatory setting (GP6). Another major disadvantage is that fewer resources are available compared to a hospital setting. Should instrumental examinations be necessary, this is associated with greater effort (GP1). The GPs addressed the lack of personal expertise and experience in approaching such challenges (GP1) (Also: *Individual Precondition - Qualification*). The work environment of GPs entails having limited on-site support, as exemplified by involving colleagues (GP6). The burden of bearing sole responsibility for decisions, without or with only limited chances for reassurance from colleagues or relatives, was perceived as challenging (GP9).

##### Legal context

The legal context played a prominent role in all the interviews. GPs’ explicit and implicit remarks showed that they perceived a lack of comprehensive legal protection when providing care to patients with palliative care needs, especially when it came to assisted suicide. This legal ambiguity has the potential to induce distress. Their own values and knowledge factor into this context (Individual Precondition). As these factors are difficult to sort from each other, they are presented together in this section. In the perception of the legal situation of physician-assisted suicide, the GPs’ assessments varied from “*illegal*” (GP3; GP11) to “*allowed under certain conditions*” (GP7). Their willingness to accommodate the request for physician-assisted suicide also varied from “*strict refusal*” (GP3; GP9; GP11) to “*providing information about alternative options*” (GP4) or to contact specific assisted dying associations (GP6). With a good and long doctor-patient-relationship, GPs were rather open to further measures: “*I think it would very much depend on whether I have accompanied the patient until then and have such a*,* already such a relationship*,* you know. […] Then I believe*,* I could imagine it more easily.*” (GP8). As inhibiting factors GPs cited personal values (GP3; GP9) and religious reasons: “*we as humans [are] not empowered to end a life*,* you know. Even if the person wants or wishes it*.” (GP11). GPs also stated role conflict, as to them physician-assisted suicide conflicted with the professional role of a GP (GP8; GP10). A significant barrier was the legal uncertainty among GPs (GP6; GP9): “*And if the patient had then asked me to put an end to it*,* then […] I would actually have been afraid of losing my profession if I did something that is not currently allowed*.” (GP10). However, the uncertainty also brought a temporarily relieving aspect: “*I’m actually glad that it’s not legally clarified yet because I can hide behind it. If it were clarified*,* I would have to deal with it*.” (GP9).

##### Social relationships

Several participants emphasized the pivotal role of emotional and social support, especially in palliative care (GP2; GP3; GP7; GP9; GP10). A stable relationship with the patients is beneficial: “*knowing the entire […] environment of the patient. […] one can assess a bit where there are problems within the family or even in the household. […] So*,* I think that is an advantage that colleagues from other disciplines cannot have because they are not so on-site. And often*,* they don’t know the patients for as long*.” (GP3). Conversely, palliative care poses a significant challenge when a stable relationship has not yet been established (GP2; GP7). The sense of security conveyed to patients with an established trust was seen as a factor that strengthens GPs in their work: “*Because I simply wanted to be there for my patients when they go. Because they have a bit more security there. After all*,* those were unfamiliar doctors [in the hospital]. […] when your own GP stands beside and essentially approves of what is happening*,* that’s something completely different.*” (GP10). When relatives or close individuals are involved in palliative care, the GPs must gain their trust as well (GP2; GP9). Gaining trust often requires (time-)intensive and emotionally charged conversations (GP5; GP6). Nevertheless, GPs considered involving relatives closely and early as an important aspect (GP9; GP11) as relatives can also reduce the workload for GPs by being guided, for example, in the administration of medications (GP11).

##### Societal conditions

The societal discourse surrounding the topic of mortality is perceived as challenging. Death is “*naturally tabooed in society*” (GP11) less so in the professional context (GP8). The societal taboo around deterred GPs from initiating these conversations (GP11). GPs even feared societal condemnation as “*involuntary angels of death*” (GP7) if they fulfill the requests for physician-assisted suicide. At the same time, there is a growing societal acknowledgment of palliative medical practices as supportive. Particularly, the gratitude expressed by those immediately affected was considered as “*justifying the effort*” (GP9).

#### Individual preconditions

##### Personal attitude

Although palliative care is an inherent responsibility, death itself played a rather subordinate role in the everyday thoughts of the GPs surveyed. In the short survey, 72.7% stated that they do not think about the death of themselves, loved ones, or patients more than once a day (Table 2). There was also predominantly little fear of contact with death. Despite the GPs’ reservations about physician-assisted suicide, they understood patients’ desire for medical support about a self-determined death (GP6; GP7; GP10, Table 2). In the survey, six GPs agreed that everyone should have the freedom to decide for themselves how they want to die.

**Table 2:** Selection of relevant questions regarding the GPs’ attitude towards death

Question	0 *n* (%*)	1 *n* (%*)	2 *n* (%*)	3 *n* (%*)	4 *n* (%*)	5 *n* (%*)
I am frequently reminded of death in my day-to-day work	0 (0)	0 (0)	2 (18,19)	3 (27,28)	2 (18,19)	4 (36,36)
You only have to think about death when you are old	6 (54,54)	4 (36,36)	1 (9,09)	0 (0)	0 (0)	0 (0)
It scares me that I myself will have to die one day	2 (18,19)	3 (27,28)	3 (27,28)	3 (27,28)	0 (0)	0 (0)
I am glad that I will die one day	2 (18,19)	3 (27,28)	3 (27,28)	1 (9,09)	2 (18,19)	0 (0)
I am afraid of my own death because it will cause pain for my familiy and friends	0 (0)	3 (27,28)	1 (9,09)	3 (27,28)	3 (27,28)	0 (0)
Everyone deserves the freedom of deciding for themselves how and when they choose to die *Missing: n = 1*	0 (0)	2 (18,19)	0 (0)	2 (18,19)	2 (18,19)	4 (36,36)
Seeing a dying person repulses me	6 (54,54)	3 (27,28)	0 (0)	1 (9,09)	0 (0)	0 (0)
Being in the presence of a dying person scares me	5 (45,45)	3 (27,28)	1 (9,09)	2 (18,19)	0 (0)	0 (0)
The sight of a dead person scares me	7 (63,63)	3 (27,28)	0 (0)	1 (9,09)	0 (0)	0 (0)
Touching someone who has recently died scares me	8 (72,72)	1 (9,09)	2 (18,19)	0 (0)	0 (0)	0 (0)

Legend: 0 = does not apply at all, 5 = applies very strongly

* Due to the small sample size (N = 11),* the values do not always add up to 100%.*

GPs found it challenging when patient or family preferences did not align with their assessment (GP8; GP11). This often occurred when family members sought maximum care, seen by GPs as an unnecessary burden.

##### Qualification

The majority of GPs (10 out of 11) felt emotionally prepared for the care of patients with palliative care needs. The level of experience in the sample was very heterogeneous. Some GPs (*n* = 4) stated in the interview that they had not attended any further training. Mostly these GPs (but not exclusively) reported a lack of knowledge (GP8; GP11). For the other GPs, the further training varied between self-study (*n* = 1), seminars (*n* = 2) and additional training in palliative medicine (*n* = 4). Despite GPs expressing a high level of clinical experience, they wanted more training, as palliative care “*is somewhat overlooked in training despite its elevated priority*” (GP3). The perceived lack of qualification increases the risk of a stress reaction. No predominant motivations for pursuing more extensive training could be discerned within the sample.

When assessing the GPs’ concerns about making mistakes in connection with palliative care, the overall mood was fairly balanced, with a slight tendency towards fear of making mistakes (Table 3).

**Table 3:** Selection of relevant questions regarding the GPs’ qualification

Question	0 *n* (%*)	1 *n* (%*)	2 *n* (%*)	3 *n* (%*)	4 *n* (%*)	5 *n* (%*)
Doing something the wrong way while interacting with a dying person worries me	3 (27,28)	2 (18,19)	-	3 (27,28)	3 (27,28)	-
I know how to listen to a dying person	-	1 (9,09)	-	3 (27,28)	2 (18,19)	5 (45,45)
I am capable of holding conversations with people who are dying	-	-	-	3 (27,28)	3 (27,28)	5 (45,45)
Having to tell someone that they are going to die soon scares me	1 (9,09)	3 (27,28)	2 (18,19)	3 (27,28)	1 (9,09)	1 (9,09)

Legend: 0 = does not apply at all, 5 = applies very strongly

* Due to the small sample size (N = 11),* the values do not always add up to 100%.*

There were discrepancies between the quantitative and qualitative data regarding communicative and psychosocial skills. In the quantitative data, the GPs predominantly expressed positivity regarding their communicative competencies. Specifically, 63.64% (n = 7) asserted competence in attentive listening to individuals in the terminal phase, and 72.73% (n = 8) expressed confidence in being able to have conversations with the dying. Communicating unfavorable prognoses presented only for 18.19% (n = 2) of GPs a specific challenge. However, in the interviews, GPs described a deficiency in training related to communication skills and offering psychological support to patients and their families (GP8; GP10; GP11) as “*no one teaches you how to conduct conversations“* (GP11).

##### Emotions

GPs described palliative care as highly emotional. Some GPs mentioned that palliative care treatment pushes them to their emotional limits (GP9; GP11). Negative emotions described were a certain sense of discomfort, frustration, and helplessness (GP2; GP5; GP9; GP10). They perceived a younger age of patients and parenthood of younger children as emotionally more challenging (GP5; GP7; GP8). When they could identify themselves with the patients (e.g., through similar age or similar family situations), emotional involvement increased as the GPs were confronted by their own mortality (GP4; GP7; GP9). It is also challenging when patients or their families react defensively or deny the overall situation (GP5; GP6; GP11).

The lack of social support for the dying (e.g. during the COVID-19 pandemic) triggered negative emotions (GP11). GPs reported that the grief of the families or the fear of the patients also affects them (GP2). This is aggravated by a *“feeling of guilt”* if the care *“simply did not go well and it could have been better*” (GP4).

However, palliative care can also evoke positive emotions, mostly described when patients are well-cared for in all four aspects of palliative medicine (physical, psychological, social, and spiritual). The social support provided by family members is particularly emphasized in this regard (GP2; GP10). GPs described gratitude from patients and family members, leading to a nonspecific positive feeling (GP6; GP7). In general, positive emotions were typically triggered when GPs felt they have achieved the best possible care for patients.

### Dealing with stressors

#### Primary appraisal

Some GPs rated palliative care as neither a positive nor a negative stimulus. If the external stimuli were assessed directly as “irrelevant”, active processing and dealing with any emotions was not necessary for the respective GPs. Some GPs appraised stimuli from palliative care activities as positive and enriching (GP3; GP6; GP7). In this case, no coping strategy was necessary as there was no negative stimulus to navigate. However, contrasting opinions also emerged, indicating that certain GPs regarded (parts of) palliative care as a potentially distressing stimulus, requiring coping strategies for the situation.

#### Secondary appraisal

The secondary evaluation occurs when a stimulus is initially perceived as potentially threatening. GPs identified high resilience and personal spirituality as protective elements for compensating for initially threatening stimuli (GP4; GP6; GP7; GP8; GP10). The interconnectedness of life and death was acknowledged, with the understanding that “*death [is] a part of our lives*” (GP6). This perspective helped them to cope with stressful situations. Addressing one’s mortality and thoughts about dying also facilitated communication with patients (GP4). Additionally, the importance of maintaining a good work-life balance was emphasized for the ability to accumulate and utilize sufficient personal resources (GP4; GP6). The GPs described a kind of emotional balancing. As positive emotions dominate in palliative care, they were able to compensate for the negative feelings (GP7).

#### Coping

If negative stressors cannot be sufficiently compensated for by protective factors, a coping mechanism becomes necessary. Problem-focused and emotion-focused coping strategies were evident in the GPs’ statements. However, a few of the GPs stated that they did not have a specific coping strategy.

##### Problem-focused coping

Some GPs described that they either proactively avoid situations that could lead to negative stressors or, at the very least, attempt to delegate a significant portion of the responsibility to other colleagues or external teams (such as SAPCT teams) (GP5; GP8, GP9). Another way to engage in problem-focused coping is through an organized work routine. Measures perceived as supportive include scheduling and incorporating buffer times (GP10) and conducting in-depth conversations to address the concerns of patients and their families. Establishing a 24-hour telephone availability for patients with palliative care needs and their families was discussed rather differently, either as supportive or as a further stressor (GP2; GP9). SAPCT teams were often cited as the primary source for both temporal and professional relief, especially as they are available 24 h a day (GP1; GP4; GP5; GP8; GP9; GP11). The lack of personal resources can thus be compensated for through interprofessional collaboration (GP5; GP7; GP9; GP11). Simultaneously, collaboration always involves an interface. Therefore, effective communication is essential, and its absence was otherwise perceived as a burden (GP7).

##### Emotion-focused coping

The spectrum of emotion-focused coping is diverse, individual methods range from practicing autogenic training (GP9) and engaging in sports (GP4) to having intensive conversations. Balint groups or colleagues in the practice were emphasized as helpful: “*One often discusses this with colleagues. I find that always super important*,* yes. […] That is actually the most effective coping measure for me*.” (GP5). GPs only occasionally mentioned conversations with family and friends as a personal relief (GP7; GP8). Individual reflection and recapitulation of situations were also described as coping strategies (GP2; GP5). GPs mentally went through the treatment situations, evaluating where things went well and identifying areas for potential improvement. The consensus between the measures taken and the wishes and needs of the patients is particularly relieving in retrospect.

Occasionally, GPs kept their thoughts and emotions at a distance, leading to expressions like *“to a certain extent*,* I don’t care”* (GP1). For some, the distancing is the only way to fulfill the role as GP and to be able to handle the emotions of patients and their families.

### ACP in primary care

Except for three GPs (GP1; GP3; GP7), the concept of ACP was unfamiliar to German GPs. The responses to ACP, however, were largely positive. Particularly, the initiation of discussions can be made easier by a structured program like ACP (GP2; GP9). Once the conversation has started, further discussions become easier: “*It’s like pushing a car. It requires a lot of effort to get it moving initially*,* but once it’s in motion*,* it’s relatively easy to push it a bit faster*.” (GP9).

The GPs perceived the concept as more detailed and specific than an advance directive, yet they believed that it does not guarantee the discussion of the actual health conditions that may occur in advance (GP4). They emphasized the time problem again (GP11), making it impractical to offer ACP to all patients (GP8). The most frequently cited reason for individual relief for GPs was the existence of a specific action plan in the event that the respective patients become incapable of making decisions (GP2; GP6; GP7; GP8; GP10; GP11), especially in acute situations (GP8). GPs also mentioned personal protection against accusations from relatives (GP7). They evaluated ACP as emotionally challenging, especially when measures need to be withheld that are not in line with their preferences (GP1; GP8). Additionally, limited relief options were perceived when the treating GPs are not part of the advance planning process (GP1).

## Discussion

### Main results

GPs regarded palliative care as an essential component of their work. They described significant challenges, with daily time constraints being the most prominent obstacle. There is a perceived lack of expertise in several aspects of palliative care. Despite difficulties, GPs expressed a commitment to enabling patients to die at home. Societal taboos often hindered discussions about death. Most GPs waited for their patients to initiate the topic. Implementing treatment limitations within palliative care was seen as a routine aspect. Their willingness to accommodate the request for physician-assisted suicide ranged from none to the willingness to take organizational measures regarding contact with assisted dying associations. Some GPs viewed aspects of palliative care as potentially distressing, requiring individual coping strategies. ACP was relatively unfamiliar among German GPs but was recognized as a valuable framework for discussing end-of-life care.

### In context with other studies

GPs stated that palliative care is part of their job confirming previous studies [25]. Both, the number of services and the utilization of palliative care, have shown consistent growth in the last years in Germany [26]. Time constraints as the most prominent barrier [27] might be intensified by the increasing number of GPs who work part-time [28]. In alignment with our findings, other studies have also suggested that the work setting serves as a risk factor. For instance, working in a palliative care unit has been linked to a lower risk of burnout compared to working in mobile palliative care teams [29], nurses in specialized palliative care have reported higher levels of satisfaction compared to those in general palliative care [30].

Some GPs expressed fear of taking away hope and, consequently, quality of life when addressing death. However, studies have demonstrated that proactive care planning can improve quality of life [31]. In patients with metastatic cancer, discussing available care options and engaging in ACP resulted in improved emotional and mental quality of life [32]. Early discussion of the final stages of life and palliative care involvement could potentially extend survival time while improving the overall quality of life [33].

A lack of knowledge was mainly reported by younger GPs which was also found in other studies [25, 27]. The participating GPs indicated that palliative care was neglected in their medical training. A review of educational curricula of medical schools in the United States identified a lack of incorporating palliative care into medical training [34]. Another study showed that despite the expansion of palliative care training programs, the evidence regarding their efficacy is poor [35]. In other studies, the impact of qualification levels on the perceived burden appeared to be minimal [36]. Nurses with higher qualifications experienced even higher levels of stress compared to those with lower qualifications [30]. The lack of knowledge may not necessarily impact the experience of stress. However, it could affect patient care as information deficits, fear, and uncertainty lead to more hospital admissions [37]. Declining end-of-life hospitalization in a medical advance care planning document can reduce the likelihood of hospitalization [38].

To some extent, GPs should display empathy in treatment situations. However, concerning their psychological well-being, there must be sufficient opportunities to acknowledge, perceive, and process these feelings. If this does not happen, distress can lead medical professionals to distance themselves more from patients or experience emotional numbness (compassion fatigue) [39–41]. Indicators of this could be identified in the interviews. Strategies against compassion fatigue, such as meditation programs can be helpful [42, 43]. Additionally, the exchange between colleagues plays an important role in ensuring empathetic care [44]. A possible strategy to alleviate distress could be the so-called “Death Cafés“. This concept, where individuals casually exchange thoughts on death and related subjects over coffee and cake, has been previously offered, specifically for healthcare personnel as well [45].

The GPs believed that the most significant relief could be achieved when they engage in ACP conversations. In Germany, however, with training various medical professional groups can conduct ACP discussions [16]. Hence, there is a need for future discussions on how to involve GPs in this process so that they can benefit from potential relief.

### Strengths and limitations of this study

The combination of both, survey and interviews, facilitated a more in-depth exploration of psychological stress experienced by GPs in their care of patients with palliative care needs. Combining two existing stress models *Stress-Strain-Model* [22] and *Transactional Model of Stress and Coping* [20] made it possible to link stressors and coping mechanisms and develop a theory-driven category system for data analysis.

We could not reach the number of participants as planned. Nevertheless, we assume that our heterogeneous sample led to a broad range of results and did not need to form any further new categories after eight interviews, indicating data saturation. The study may have predominantly included GPs with a particular interest in this topic (recruitment bias). The title, which referred to “psychological stress” may have contributed to the fact that not all GPs felt addressed. The focus of this research was psychological stress in the context of external and internal factors. We did not look at stress as a biological factor. Some of the GPs classified the stimuli arising from palliative care as “irrelevant”. This may already represent a protective mechanism for the GPs. An exact differentiation between “irrelevant” and protective mechanisms was not possible based on the interviews. Furthermore, we assumed that ACP is unknown among GPs which may have influenced the results.

## Conclusions

Palliative care can be associated with negative psychological stress for GPs. However, the actual work with patients with palliative care needs is not always the challenging aspect; often, the challenging issues arise from the surrounding conditions, such as inadequate billing options, time constraints, or uncertainties regarding legal matters. Despite individual coping strategies already in place, it is advisable to explore concepts for professional psychological relief. It is crucial to increase awareness of ACP’s existence and offer training opportunities. A clearer legal framework must be created and communicated to GPs in an easily accessible manner by the legislator.

### Electronic supplementary material

Below is the link to the electronic supplementary material.

Supplementary Material 1

## Data Availability

The full datasets used and analysed are available from the corresponding author upon reasonable request.

## Declarations

### Authors’ information

The present work was performed in (partial) fulfillment of the requirements for obtaining the degree “Dr. med.” for VL at the Friedrich-Alexander-Universität Erlangen-Nürnberg (FAU).

## Abbreviations

ACP: Advance Care Planning
GP: General Practitioner
SAPCT: Specialized Ambulatory Palliative Care Teams
