# Supplementary material for: Psychological stress of general practitioners in the care of patients with palliative care needs: an exploratory study
Source: BMC Palliat Care. 2024 Aug 3;23:197. doi: 10.1186/s12904-024-01529-w (PMC11297742; doi:10.1186/s12904-024-01529-w)
Supplement: Supplementary file 1 — Supplementary Material 1 [file 12904_2024_1529_MOESM1_ESM.docx]

# Supplemental Information 1

**Interview Guide**

**1. Recalling the last death of a patient**

*„Today I would like to talk to you about dealing with dying/palliative patients, about the emotions it triggers in you and the impact it has on you personally. Please start by telling me about the last case of death among your patients.“*

- When was the last time a patient died?
- What caused the patient's death?
- How long did you care for the patient?
- Were you present at the time of death?
- Was there a patient's advance directive/ were personal preferences regarding the patients passing clearly communicated?
- Did you take/ refrain from taking measures to simplify the process of dying? If yes, which measures did you take?

**2. Palliative care as a GP**

Have you taken part in any palliative care seminars/ training? If yes, can you pinpoint a reason? If no, can you pinpoint a reason?

- What kind of measures that you usually implement in the care of patients with an unfavourable prognosis would you classify as palliative care?
- Which role does palliative care play in your everyday work?
- Which factors do you consider to be an obstacle in providing satisfactory palliative care?
- Which factors do you consider to be beneficial in providing satisfactory palliative care?

**3. Addressing of well-considered death wishes and surrounding emotions**

When dealing with patients experiencing palliative situations, it is increasingly common for them to address wishes regarding their own death. In this context, GPs are occasionally asked to provide assistance in terminating the patient’s life, to limit treatment or to provide medical support surrounding the patient’s death wish. Have you ever had patients ask you to provide this kind of assistance?If so, how frequent is the occurrence of such requests? What kind of feelings are triggered by such requests?

- Which of these following measures have you already taken in the care of palliative patients?
- Counselling
- Referral to an organization for assisted suicide
- Providing medication to enable suicide to be carried out in accordance with the patients well-considered wishes
- Providing expert reports for organizations as a prerequisite for carrying out an assisted suicide
- How did you feel after taking such measures?
- Did you feel legally protected when taking (or refusing) measures, or did you feel rather uncertainty (regarding the legality)?

**4. Emotions experienced in (immediate) palliative care**

What kind of emotions have you personally experienced while being involved in the care of dying patients?

- If so, please elaborate, what you experience as straining precisely?
- Death/ dying in general?
- Aggravation due to closer relationship between physicians and patients?
- Conflicts with relatives etc. if there are no specific instructions by the patient regarding their wishes?
- Powerlessness, as death is inevitable?
- Limits of palliative therapy in symptom relief?
- Legal uncertainties regarding the legality of the measures taken?
- Demand for constant availability?
- Time pressure + integration of such "complex patients" into everyday working life
- Lack of compensation for the increased effort?
- Are there moments in which you perceive palliative care as an enrichment? If so, which moments in particular?
- Facilitating the dying of suffering patients
- Care for long-term patients until the end
- Sense of security for patients and their family members if you are present/available as a GP
- Certainty that everything in your power has been done
- What do these feelings depend on?
- Age of the patients
- Type/ duration of illness
- Duration of knowing the patient

**5. Dealing with potential psychological stress/ burdens**

- Which personal coping strategies do you employ?
- Conversations with family/ friends
- Conversations with colleagues
- Conversations with psychologists/ professionally trained staff
- Spiritual practices
- Distraction (sport, hobbies, ...)
- Do you feel that talking about these issues is considered a "taboo subject"?
- Do you feel left alone in such (supposedly stressful) situations (e.g. because there are usually no immediate colleagues present)?
- Have you ever thought about changing your field of occupation due to these psychological burdens? If so, where to?

**6. Suggestions for improvement**

Do you currently see a fundamental need for improvement in dealing with psychological stress in the care of palliative patients from the perspective of GPs? Can you think of any specific suggestions to make it easier to deal with the subject of death and dying?

**7. Advance care planning**

Do you have conversations with your patients about death/dying and what their wishes are in this regard?

- If yes:
- When/on which occasions do you have these conversations?
- Who initiates these conversations?
- What feelings do conversations like this trigger in you?
  - Relief?
  - Strain?
  - Discomfort?
  - Stress (e.g. due to lack of time)
- What do you think about the amount of time needed for such discussions?
- Do you have enough time in your normal working day to have conversations like this?
- Do you feel you have a responsibility to talk to your patients about such matters?
- If not:
- Can you think of a reason why not?
- Is it a conscious decision, or has the topic not yet come up?
- Do you feel it is not your area of responsibility?
- Do you feel you don’t have enough time?
- Is it because there is no good way of billing?
- Have you ever heard of the term "Advance care planning"?
- If so, what do you understand by this term? Can you try to define the term?
- Do you think that regularly addressing the topic of death from your side could be helpful in order to relieve you in the "acute situation"?

Thank you very much for the detailed and informative conversation and your personal views on this topic. Are there any additional points you would like to add?

# Supplemental Information 2

| **Question** | **Answers & Frequency** |
| --- | --- |
| Do you think about the death of the following groups more than once a day?  *Multiple answers possible* | - Yes, about the death of patients (n = 3) - Yes, about the death of persons close to me (n = 1) - Yes, about my own death (n = 1) - No (n = 8) |
|  |  |
|  |  |
|  |  |
| Do you feel that you have the expertise to care for patients in palliative situations? | - Yes (n = 8) - No (n = 2)   *Missing: n = 1* |
| Do you feel that you are emotionally capable of caring for patients in palliative situations? | - Yes (n = 10) - No (n = 1) |
| How many palliative patients do you care for in a year on average? | - 0 – 20 (n = 8) - 21 – 40 (n = 1) - 41 – 60 (n = 1) - >60 (n = 1) |
| How many patients with palliative care needs pass away in your care over the span of one year (approximately)? | - 0 – 20 (n = 10) - 21 – 40 (n = 0) - 41 – 60 (n = 1) - >60 (n = 0) |
| Have you heard of the term "Advance Care Planning" before learning about this study? | - Yes (n = 3) - No (n = 8) |

**Selected questions from the Questionnaire on attitudes towards dying, death, and the afterlife (FESTD)**

*Please answer by choosing the extent to which the following statements apply to you, and/or from your point of view (0= does not apply at all, 5 = applies very strongly). The term 'dying' refers to people whose end of life is imminent.*

| **Question** | **0**  **n**  **(%*)** | **1**  **n**  **(%*)** | **2**  **n**  **(%*)** | **3**  **n**  **(%*)** | **4**  **n**  **(%*)** | **5**  **n**  **(%*)*** |
| --- | --- | --- | --- | --- | --- | --- |

| 1. I am frequently reminded of death in my day-to-day work | 0  (0) | 0  (0) | 2  (18,19) | 3  (27,28) | 2  (18,19) | 4  (36,36) |
| --- | --- | --- | --- | --- | --- | --- |
| 1. You only have to think about death when you are old | 6  (54,54) | 4  (36,36) | 1  (9,09) | 0  (0) | 0  (0) | 0  (0) |
| 1. It scares me that I myself will have to die one day | 2  (18,19) | 3  (27,28) | 3  (27,28) | 3  (27,28) | 0  (0) | 0  (0) |
| 1. I am glad that I will die one day | 2  (18,19) | 3  (27,28) | 3  (27,28) | 1  (9,09) | 2  (18,19) | 0  (0) |
| 1. I am afraid of my own death because it will cause pain for my familiy and friends | 0  (0) | 3  (27,28) | 1  (9,09) | 4  (36,36) | 3  (27,28) | 0  (0) |
| 1. Everyone deserves the freedom of deciding for themselves how and when they choose to die   *Missing: n = 1* | 0  (0) | 2  (18,19) | 0  (0) | 2  (18,19) | 2  (18,19) | 4  (36,36) |
| 1. I know how to listen to a dying person | 0  (0) | 1  (9,09) | 0  (0) | 3  (27,28) | 2  (18,19) | 5  (45,45) |
| 1. Doing something the wrong way while interacting with a dying person worries me | 3  (27,28) | 2  (18,19) | 0  (0) | 3  (27,28) | 3  (27,28) | 0  (0) |
| 1. Seeing a dying person repulses me | 6  (54,54) | 4  (36,36) | 0  (0) | 1  (9,09) | 0  (0) | 0  (0) |
| 1. Being in the presence of a dying person scares me | 5  (45,45) | 3  (27,28) | 1  (9,09) | 2  (18,19) | 0  (0) | 0  (0) |
| 1. Having to tell someone that they are going to die soon scares me | 1  (9,09) | 3  (27,28) | 2  (18,19) | 3  (27,28) | 1  (9,09) | 1  (9,09) |
| 1. I am capable of holding conversations with people who are dying | 0  (0) | 0  (0) | 0  (0) | 3  (27,28) | 3  (27,28) | 5  (45,45) |
| 1. The sight of a dead person scares me | 7  (63,63) | 3  (27,28) | 0  (0) | 1  (9,09) | 0  (0) | 0  (0) |
| 1. Not being able to do anything about a person's death scares me | 3  (27,28) | 4  (36,36) | 1  (9,09) | 1  (9,09) | 2  (18,19) | 0  (0) |
| 1. Touching someone who has recently died scares me | 8  (72,72) | 1  (9,09) | 2  (18,19) | 0  (0) | 0  (0) | 0  (0) |

*Due to the small sample size (**N = 11)**, the values do not always add up to 100 %.

# Supplemental Information 3

Excerpt from Coding Tree

| **Main Category** | **Sub Category** |  | **Description** |
| --- | --- | --- | --- |
| **Role of palliative care** | Low |  | Palliative medicine has a relatively low priority in their current workload. |
|  | High |  | Palliative medicine has a relatively high priority in their current workload |
| **External stressors** | Job content | Measures | Participants describe which medical measures are associated with palliative medicine |
|  |  | Demand for support during the dying process | Who is asking? When is it discussed? How is it reacted to? |
|  | Work environment | Palliative care situation | The advantages of outpatient palliative care resulting from healthcare system-related factors are mentioned or care situations at home |
|  |  | Outpatient setting | Outpatient setting and the associated conditions that are perceived as hindrances |
|  | Legal context | General perception of the legal situation | Description to what extent they feel legally protected when taking or refraining from measures within the scope of palliative medical care. ALSO granted, when personal measures (e.g., further education) are described to enhance legal protection. NOT granted, when the participant speaks about the lack of legal legitimization of physician-assisted suicide, which is why it is not carried out by the participants. |
|  | Social relationships | Lack of doctor-patient relationship | when the doctor-patient relationship is described as brief or not yet sufficiently intensive, resulting in challenging patient care. |
|  |  | Stable doctor-patient relationship | Favorable aspects resulting from a stable doctor-patient relationship |
|  |  | Differences in expertise versus wish | When it is perceived as hindering/problematic/stressful if treatment wishes do not align with the medical expertise of the GP |
|  |  | Patients' preferences | When it is considered beneficial by the participants to fulfill the patients' wishes |
|  | Societal conditions | Taboo topic | Death is a taboo topic |
|  |  | Societal recognition | Growing societal recognition of palliative medical work is perceived as supportive |
|  |  | Societal approach | When difficulties arising from societal attitudes towards death/dying/palliative care are mentioned |
|  | Work tools | Deficient billing | Participants do not perceive the financial remuneration as sufficient or adequate |
|  | Work organisation | Time management | Participants perceive good time management as beneficial |
|  |  | Organizational difficulties | Problems in time/schedule management |
|  |  | Good collaboration with relatives | Collaboration with relatives is perceived as beneficial |
|  |  | Good collegial support/Network | Collaboration with relatives is described |
|  |  | Care structures | When participants mention structures and resources in care that can be accessed |
|  |  | Communication difficulties | Difficulties in communication with other involved professional groups or with patients/family members |
|  |  | Deficient planning | When the fact that no medical planning has been documented by the patients is perceived as hindering/making care difficult. |
| **Individual Preconditions** | Personal attitudes | Personal attitude toward self-determined death | When the personal attitude towards self-determined death is described or when participants imagine themselves in the role of a patient. |
|  | Qualification | Need for further education | To what extent there is a need for further education in palliative medicine |
|  |  | Expertise | Training in the field of palliative medicine is perceived as supportive, further training has not yet been completed, but participants believe they would be supported by it and when previously gathered personal experiences are perceived as supportive |
|  |  | Lack of knowledge | Participants mention that difficulties may arise due to lack of experience/knowledge |
|  | Emotions | Emotions in palliative care | Participants describe which emotions they have already observed within themselves while being involved in general palliative care |
| **Dealing with stressors** | Primary Appraisal | positiv | Palliative medical work is generally perceived as a positive stimulus |
|  |  | dangerous | When participants perceive palliative medical work as a challenge, threat, or harm. Also granted, when participants are aware of colleagues interpreting it as such |
|  |  | irrelevant | By maintaining a professional distance, emotions are kept separate from the role as a physician or participants intentionally keep potentially stressful situations out of conscious awareness, thus rendering the situation irrelevant |
|  | Secondary Appraisal | Resilience | Participants describe a high personal resilience to potentially stressful situations in palliative care. |
|  |  | Spirituality | Spirituality as a resource to cope with potentially stressful situations in palliative care |
|  |  | Work-Life Balance | A certain amount of leisure time helps to cope with potentially stressful situations in palliative care |
|  | Problem-focused coping | Organizational aspects | Organizational measures as relieving and enabling participants to better cope with stressful situations |
|  |  | Collaboration | Lack of personal resources can be compensated for through interprofessional collaboration |
|  | Emotion-focused coping | Collegial exchange | Conversations with colleagues are mentioned as a coping strategy |
|  |  | Family/friends | Conversations with family/friends are mentioned as a coping strategy |
|  |  | Assessment of situations/emotions | Situations/emotions are reflected upon and assessed. |
|  |  | Physical activity | Physical activity is used as a coping strategy |
|  |  | Autogenic training | Autogenic training is used as a coping strategy |
| **Advance Care Planning** | Knowledge |  | Knowledge about ACP, its goals |
|  | Advantages |  | Possible advantages of ACP |
|  | Disadvantages |  | Possible disadvantages of ACP and suggestions for the future |
